# Evaluating self-medication practices in Ethiopia

**DOI:** 10.1186/s40545-023-00553-0

**Published:** 2023-03-21

**Authors:** Yabibal Berie Tadesse, Abebe Tarekegn Kassaw, Eyayaw Ashete Belachew

**Affiliations:** 1 Pharmaceutical Chemistry Department, School of Pharmacy, College of Medicine and Health Sciences, University of Gondar, Gondar, Ethiopia; 2 Department of Pharmacy, College of Medicine and Health Sciences, Woldia University, Woldia, Ethiopia; 3 Department of Clinical Pharmacy, School of Pharmacy, University of Gondar, Gondar, Ethiopia

**Keywords:** Self-medication practice, Antibiotics, Adult patients, Outpatient department, Public hospitals, Addis Ababa

## Abstract

**Background:**

Self-medication with antibiotics has become an important factor driving antibiotic resistance and it masks the signs and symptoms of the underlying disease and hence complicates the problem, increasing drug resistance and delaying diagnosis. This study aimed to assess the extent of self-medication practice with antibiotics and its associated factors among adult patients attending outpatient departments (OPD) at selected public Hospitals, in Addis Ababa, Ethiopia.

**Methods:**

Facility-based cross-sectional study was employed. A systematic random sampling technique was used to include the study participants. Self-administered with structured questionnaires were applied among patients who visited outpatient departments at selected public Hospitals, in Addis Ababa. Data were entered into Epi-data version 4.6 and analyzed using SPSS version 26. Descriptive statistics such as frequencies and percentages were used for the present categorical data. The data are presented in pie charts, tables, and bar graphs. Furthermore, bivariable and multivariable binary logistic regression analyses were used to identify significant associations. Statistical significance was declared at *p* value < 0.05.

**Results:**

Out of 421 respondents interviewed, 403 (95.7%) participants completed questionnaires. Among the respondents, 71% had generally practiced self-medication. Among these, 48.3% had self-medication with antibiotics, while 51.7% had used other drugs. The most commonly cited indication for self-medication practice was abdominal pain 44.9%, followed by Sore throat 21% commonly used antibiotics are amoxicillin (57%), ciprofloxacin (13%), amoxicillin/clavulanic (10%), erythromycin (8%), cotrimoxazole (7%), and doxycycline (5%).

**Conclusions:**

Self-medication with antibiotics was common among the study participants. The prevalence of general self-medication was 71%, whereas that of self-medication with antibiotics was 48.3%. In general, the potentially dangerous effects of SMP seem to be underestimated by patients with OPD patients.

## Introduction

Self-medication has traditionally been characterized as using medications, or home remedies on one’s own initiative or at the suggestion of another person without first contacting a medical professional [1]. The World Health Organization (WHO), defined self-medication as the selection and use of drugs to address self-identified diseases or symptoms. Self-medication is when a person obtains and consumes a medication without seeking medical assistance, whether for diagnosis, prescription, or monitoring of one’s own therapy or medication [2].

It usually involves over-the-counter (OTC) medications, but it can also include prescription-only medications (POM, purchasing drugs by reusing or resubmitting a previous prescription, taking medications on the advice of a relative or other, or eating leftover medications already on hand at home [3]. The FDA (2006) characterizes OTCs as a sedate item promoted for use by the buyer without the intercession of a well-being care proficient in arranging to get the item. With respect to the classification of drugs, it appears that individuals do not separate between medication and OTC drugs [4].

Prescription products are medications that require a doctor’s prescription [5]. Several studies conducted in indicated that self-medication with antibiotics is quite common, varies by community and social determinants of health, and is usually accompanied by the use of unsuitable drugs [6]. In developed countries, the use of antibiotics without prescription is the second highest prevalent next to antipain [7]. Antibiotics are not available over the counter, and they require a prescription before being dispensed. Over the counter products are also available at supermarkets and other community pharmacies in various countries, including Ethiopia [8].

Inappropriate self-medication waste resources raises the risk of drug resistance and leads to major health issues such adverse drug responses, treatment failure, prescription misuse, and drug addiction [9]. Despite this, self-medication may save money on health care and time spent waiting to visit a doctor for mild diseases.

Self-medication has several detrimental effects on one’s health. It may result in incorrect self-diagnosis and a delay in receiving urgent medical care. Moreover, it may lead to ineffective dosing, polypharmacy, and hazardous drug interactions [10]. This can lead to noncompliance with a drug regimen that can result in serious outcomes such as adverse drug reactions and reduction in the quality of treatment [10]. Moreover, currently, there is a worldwide concern about the emergence of antibiotic-resistant strains of micro-organisms, which might have been highly augmented by self-medication [11].

Self-medication has been reported to be on the rise around the world and has become a public health concern [9]. People in poor countries are self-medicating with not only non-prescription but also prescription medications without supervision. Although the WHO has stressed the importance of properly teaching and controlling self-medication, its use is nevertheless widespread [12].

A number of researches on various elements of self-medication have been undertaken internationally, and the prevalence of self-medication among adult outpatients has been found to be high [13–18]. The prevalence of self-medication in Greece was 77.9% in [19], 98% in Palestine [20], 71% in India [21], and 76% in Pakistan [21]. The rates are similar in Africa: It is 99.4% in Nigeria [22], 56% in Malawi [23], 53.5% Kenya [24], 75.7% in Uganda [25] and 50% in Ethiopia [8]. Accordingly, individuals practiced self-medication for different purposes. Studies have reported that headache, fever, cough, gastrointestinal diseases, respiratory tract infections, maternal/menstrual, eye diseases, skin diseases, injury, and sexually transmitted diseases were common indications for self-medication practice [26].

A few studies have been undertaken in Ethiopia to investigate the usage of self-medication among the public and students, including medical students [27–29], and there are indications on the misuse of antibiotics by patients, even by health professionals [30]. However, there is no study conducted on self-medication practice with antibiotics of adult outpatients in Ethiopia. The findings of this study will fill the research and knowledge gap. In addition, this study will generate information that may be useful in policy development and review of policies on licensing of drugs. Furthermore, it can be used as a stepping stone for health professionals if there is any possibility of intervention. Finally, this research can be used as a base for other health professionals including pharmacists in understanding the situation of the case and extending their intervention or work to different institutions also the findings potentially assisting in the development of appropriate regulatory and administrative solutions in Ethiopian hospitals. As a result, this study aimed to assess self-medication practice with antibiotic-among adult patients in OPD at selected public hospitals in Addis Ababa, Ethiopia.

## Materials and methods

### Study design, setting and period

An institution-based cross-sectional study was conducted from February 2022 and March 2022, in selected public hospitals, Addis Ababa, Ethiopia. Addis Ababa is the capital city of Ethiopia, which contains 13 government hospitals (5 federals, 6 under Addis Ababa health bureau, one owned by the police force, and one owned by armed force) distributed throughout ten sub-cities. All Hospitals provide different OPD services. Four hospitals had been selected using simple random sampling by lottery method from the list of thirteen hospitals [31]. Tikur Anbessa specialized Hospital, St. Paul Hospital Millennium Medical College, Minillik II Hospital and St. Peter specialized hospital. Tikur Anbessa Specialized hospital and St. Paul’s Hospital both are the largest referral and teaching hospitals in Ethiopia and are operated under the ministry of health. St. Peter specialized Hospital is the other referral and teaching hospital among those operated under the federal ministry of health. However, Minillik II Hospital is among the six governmental referral hospitals that are managed under the Addis Ababa Administrative Health Office. Patients who attended outpatient clinics in the hospital were expected to provide information in respect to factors associated with SMA. The above selected hospitals outpatient clinics had a high volume of patients and this enabled the researcher to collect data from the subjects from the proposed sample size within the limited time of the survey. Patients visiting the hospital due to their illnesses may have had prior knowledge of SMA, unlike a person in the community who may not have experienced an illness.

### Study participants and eligibility criteria

All adult patients seeking treatment in OPD in the selected public Hospital during the study period and fulfilled the inclusion criteria were included. addition, patients attending out patients in selected PHs who were above 18 years of age, and those who had taken informed consent were included, whereas, participants who were admitted in the ward, unconscious, unable to speak and hear, critically ill patients and who were not willing to participate in the study were excluded in this study.

### Sample size determination and sampling techniques

Sample size (*n*) had been calculated on the basis of a single population proportion formula assuming that the prevalence of self-medication with antibiotics is taken from a previous similar study in Kenya to calculate the sample size 0.476 is taken [32]:

$$n = \frac{{z_{ \propto /2}^2\;p(1 - p)}}{{d^2}}$$

The assumptions used are: *z* value of 1.96 at 95% confidence interval (CI) and margin of error (*d*) is 5%, non-response rate 10%:

*d* marginal error of 5%; *p* proportion; *n* sample size.

$$n=\frac{{(1.96)}^{2}*0.476\left(1-0.476\right)}{{(0.05)}^{2}}=383.$$

**Fig. 1:**
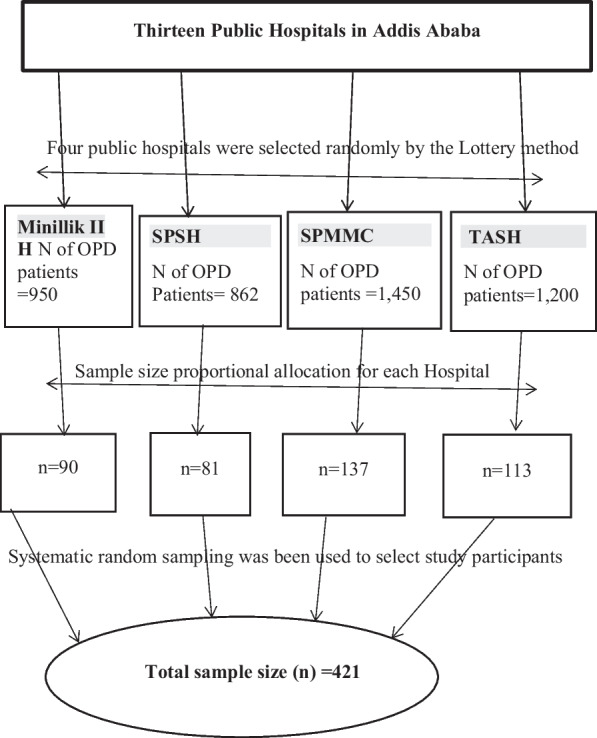
Schematic representation of the sampling procedure to select study participants from Addis Ababa selected public hospitals, 2022

By adding 10% for incomplete and non-responses, the total sample size required for this study was found to be 421. From 13 governmental Hospitals in Addis Ababa, Tikur Anbessa, St. Paul, Minillik II, St. Peter hospitals and St. Paul’s Hospital Millennium Medical College were selected randomly using a lottery method. To select 421 participants from adult medical and surgical OPD from a total of four selected PHs, first, all selected hospitals were listed down with their respective number of OPD patients per month. A stratified sampling method was performed with the strata being outpatient department, surgical, and medical outpatient clinics. Data were taken from each hospital with of monthly OPD patients report, and then, the number of OPD patients in each hospital was proportionally allocated for sample size, and then, finally the study participants for each hospital were selected and interviewed with a systematic random sampling method in every *k*th interval of each respective hospital until the required sample size was achieved (Fig. 1).

From the past 3-monthly report from November, 2021 to January 2022 of each hospital indicate that the average number of adult OPD cases at Tikur Anbessa specialized hospital, Minillik II, St. Peter hospitals and St. Paul’s Hospital Millennium Medical College, were 1200, 950, 862 and 1450, respectively.

The total sample size (421) was allocated proportionally for the four public hospitals based on the number of OPD patients seeking treatment in a month of each hospital was: ${{n}_{j}}=\frac{{n}\times{N}_{j}}{N}$ and interval size; ${{k}_{j}}=\frac{Nj}{n}$

where *n*_*j*_ was the sample size of the *j*th hospital;

*N*_*j*_ was population size of the *j*th hospital;

*n* = nTASH + nSPMMC + *n* Minillik H + NSPSH was the total sample size (421);

*N* = NTASH + NSPMMC + NMinelikH + NSPSH was the total population size of hospitals

= 1200 + 1450 + 950 + 862 = 4462;

nTASH = 421 * 1200/4462 = 113; *k* = 1200/113 = 11, SPMMC = 421 * 1450/4462 = 137; *k* = 1450/137 = 11, Minillik II Hospital = 421 * 950/4462 = 90; *k* = 950/90 = 11, SPSH = 421 * 862/4462 = 81; *k* = 862/81 = 11.

Therefore, the initial sample was selected using the lottery metho; then, every 11th participant was selected until the calculated sample size was attained within the data collection period’s time frame.

### Variables of the study

Socio-demographic factors were age, sex, education, marital status, religion, and occupation. Behavioral and social factors such as: mild illness, prior experience, emergency use, stressful conditions, chronic illness, advice from others, laws controlling, policy factors, insurance, and knowledge on risks of use. Health facility factors are the availability of antibiotics, diagnosis, healthcare costs, ease of access, prescription, queues, and save time and attitudes, such as attitudes, distance, queues, policy factors, and laws. The dependent variable was self-medication practice with antibiotics. This was determined as the report of taking drugs without a prescription among adult outpatients.

### Data collection and procedures

The questionnaire was first prepared in the English language and translated into Amharic by a language expert for interviews. To check the accuracy and its consistency, the questionnaire was pretested on 21 participants (5% of the sample size) in one hospital in Addis Ababa, and this facility was excluded from the actual study before the start of the actual data collection period. Data were collected using structured and pretested questionnaires. Three BSc pharmacists participated in the data collection after 1 day of training were given on the objective, the relevance of the study, the confidentiality of information, respondent’s right, and informed consent. Frequent checks were made on the data collection process to ensure the completeness of principal investigators and supervisor.

### Data quality assurance

Data collectors were trained intensively by the principal investigator on the contents of the questionnaire, data collection methods, and ethical concerns. The filled questionnaire was checked daily for completeness by the principal investigator for uniformity and understandability of the checklist after which modification for its appropriateness and suitability was performed. Data collectors had trained on strict use of study criteria, an explanation of study objective, getting verbal consent from study participants, uniform interpretation of questions, and the collected data confidentiality.

### Data processing and analysis

After checking the collected data for its completeness and accuracy, codes were given to the questionnaire; then, the data were entered using Epi data 4.6 statistical software and analyzed using the SPSS version 26 statistical package. Binary logistic regression was used to determine the association between the explanatory and outcome variables, and multivariable logistic regressions were used to determine the association between dependent and independent variables, *P* value < 0.05 was considered as statistically significant.

### Operational definition

Self-medication: getting and using conventional medications for disease diagnosis, treatment, or prevention without a doctor’s prescription.

Antibiotics: this is a drug used in the treatment and prevention of bacterial infection.

Adverse reaction: this is an unwanted effect caused by administrating a drug.

Outpatient: patient who attends for treatment in an outpatient clinic without staying there overnight.

Outpatient department: is part of a hospital designed for treating outpatients for whom they have health problems but do not require a bed or to be admitted for overnight care.

Over the counter/non-prescribed drugs: are those drugs that can be legally purchased from a drug retail outlet without having a prescription from a licensed healthcare provider.

Self-medication practice: a person is said to practice self-medication if he/she self-medicated at least once [32].

### Ethical consideration

Ethical clearance was obtained from the Institutional Review Board RVU college of Health science and a support letter obtained from Addis Ababa health bureau administration for each Hospital. The objective and importance of the study was explained to the study participants; then, data were collected only after full informed verbal and written consent was obtained. The confidentiality of the information was maintained by excluding the participants’ name in the interview questionnaire.

## Results

Among 421 respondents approached for the study, 403 (95.7%) were included in the final analysis. Around two-third (69%) the respondents were females. The mean (± SD) age of the participants was 33 (± 21) years. More than one-third of the (36.7%) participants was between 25 and 34 years. The majority of the respondents (62.5%) were not employed, while those who were formally employed were 24.8%. Around three-fourth (73%) of the respondents had no medical insurance scheme (Table 1).

**Table 1:** Socio-demographic characteristics of participants who used drugs for self-medication (*n* = 403)

Characteristics	Frequency	Percentage
Age in years		
18–24	55	13.6
25–34	148	36.7
35–44	87	21.6
> 45	113	28.1
Sex		
Male	125	31.0
Female	278	69.0
Marital status		
Married	270	67.0
Never married	77	19.1
Divorced	15	3.7
Widowed	41	10.2
Occupation		
Unemployed	252	62.5
Employed		
Formally employed	100	24.8
Business	51	12.7
Religion		
Orthodox	159	39.5
Muslim	71	17.5
Protestants	173	43
Healthcare insurance use		
Yes	109	27
No	294	73

### Education levels of the respondents

**Fig. 2:**
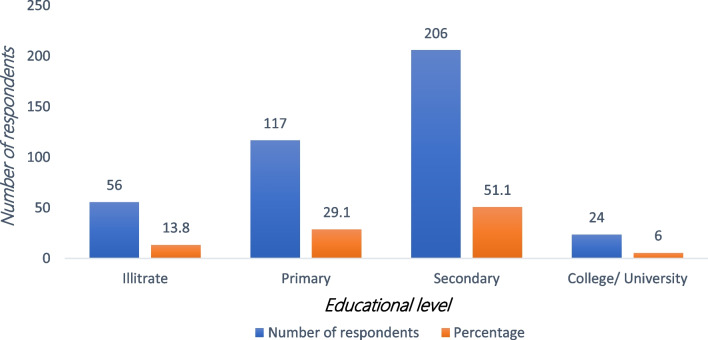
Education levels of the respondents

Education levels were categorized into four: those who had not gone to school, primary, secondary, and college/university. The respondents who had College/University were 6% (24), secondary education was 51.1% (206), those with primary education were 29.1% (117), and those who had not gone to school were 13.8% (56) (Fig. 2).

### Prevalence of self-medication

#### General prevalence of self-medication

**Fig. 3:**
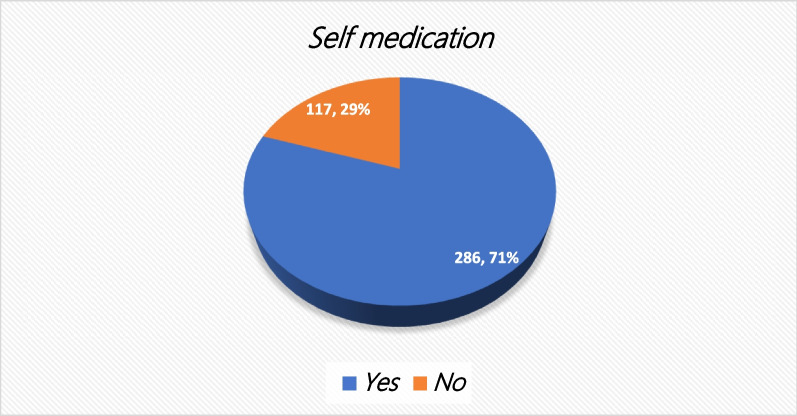
General prevalence of self-medication

The participants were asked whether they had ever taken any drug without prescription. Among the total respondents, majority of them (71%), the respondents had generally practiced self-medication (Fig. 3).

### Prevalence of self-medication with antibiotics

The respondents were required to determine whether they had ever taken any antibiotic without prescription. Around half of the participants (48.3%) had used antibiotics, while 51.7% had used other drugs (Table 2).

**Table 2:** Prevalence of self-medication use

Self-medication with antibiotics	Responses	Percentage
Yes	138	48.3
No	148	51.7
Total	286	100

#### Number of times respondents self-medicated

**Fig. 4:**
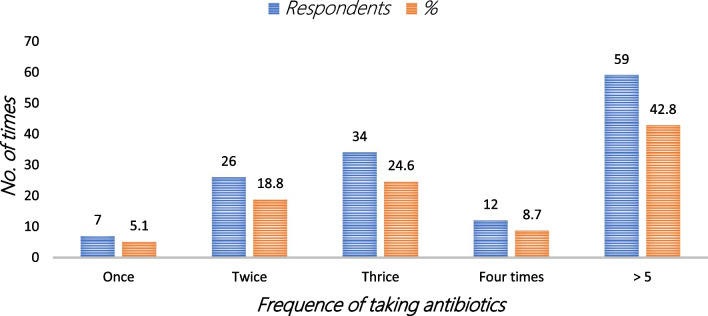
Number of times respondents self-medicated

More than half of the respondents, 59 (42.8%) had used antibiotics above five times in the past year, 34 (24.6%) three times, 26 (18.8%) twice, 12 (8.7%) four times, and 7 (5.1%) once (Fig. 4).

### Factors associated with self-medication

The age of the respondents was categorized in groups ranging from 18 to 24 years, 25 to 34 years, 35 to 44 years, and those above 45 years. Age and medical insurance scheme were significantly associated with self-medication (*P* < 0.05) in the Chi-square test (Table 3).

**Table 3:** Factors associated with self-medication

	Self-medicated	Not self-medicated	*N*	*Χ*^2^	*P* value
Age of participants					
18–24	23 (41.8%)	32 (58.2%)	55	8.697	*P* = 0.0004
25–34	46 (31.1)	102 (68.9%)	148		
35–44	32 (36.8%)	55 (63.2%)	87		
> 45	37 (32.7%)	76 (67.3%)	113		
Total	138	265	403		
Sex					
Male	51 (40.8%)	74 (59.2%)	125	0.7946	0.4782
Female	87 (31.3%)	191 (68.7%)	278		
Total	138	265	403		
Marital status					
Married	88 (32.6%)	182 (67.4%)	270	0.6380	0.3280
Single					
Never married	29 (37.7%)	48 (62.3%)	77		
Divorced	7 (46.7%)	8 (53.3%)	15		
Widowed	14 (34.1%)	27 (65.9%)	41		
Total	138	265	403		
Educational status					
Not gone to school	14 (25%)	42 (75%)	56	3.4352	0.5674
Primary	34 (29.1%)	83 (70.9%)	117		
Secondary	79 (38.3%)	127 (61.7%)	206		
College/university	11 (45.8%)	13 (54.2%)	24		
Total	138	265	403		
Occupation					
Unemployed	78 (31%)	174 (69.0%)	252	4.2760	0.643
Employed					
Formally employed	46 (46%)	54 (54%)	100		
Business person	14 (27.5%)	37 (72.5%)	51		
Subtotal	60 (39.7%)	91 (60.3%)	151		
Total	138	265	403		
Members of health insurance					
Yes	21 (19.3%)	88 (80.7%)	109	6.024	0.0176
No	117 (39.8%)	177 (60.2%)	294		
Total	138	265	403		

### Independent predictors of self-medication

In the multivariable regression model, participants who had College and university students were 1.6 times likely to practice self-medication compared with those who never went to school or illiterate [AOR = 1.65, 95% CI (1.3–2.4), *P* = 0.004]. The lack of medical insurance was also significantly associated with self-medication with antibiotics [AOR = 1.632, 95% CI (1.21–2.63), *P* = 0.033] (Table 4).

**Table 4:** Independent predictors of self-medications

Variable	SMP	None SMP	OR 95% CI	*P* value
Age				
18–24	23 (41.8%)	32 (58.2%)	1	
25–34	46 (31.1%)	102 (68.9%)	0.453 (0.51–0.67)	0.801
35–44	32 (36.8%)	55 (63.2%)	0.722 (0.731–0.82)	0.057
> 45	37 (32.7%)	76 (67.3%)	0.576 (0.60–0.84)	0.831
Sex				
Male	51 (40.8%)	74 (67.3%)	0.955 (0.874–0.973)	0.892
Female	879 (31.3%)	191 (68.7%)	0.836 (0.76–0.98)	0.61
Education				
Illiterate	14 (25%)	42 (75%)	1	
Primary	34 (29.1%)	83 (70.9%)	0.986 (0.9–1.97)	0.236
Secondary	79 (38.3%)	127 (61.7%)	1.241 (0.93–2.5)	0.438
College and above	11 (45.8%)	13 (54.2%)	1.65 (1.3–2.4)	0.004
Marital status				
Never married	29 (37.7%)	48 (62.3%)	1	
Married	88 (32.6%)	182 (67.4%)	0.687 (0.63–1.84)	0.39
Divorced	7 (46.7%)	8 (53.3%)	1.614 (1.2–2.42)	0.057
Widowed	14 (34.1%)	27 (65.9%)	0.642 (0.36–1.31)	0.634
Occupation				
Unemployed	78 (31%)	174 (69.0%)	0.984 (0.656–1.42)	0.13
Formally employed	46 (46%)	54 (54%)	1.129 (0.83–1.76)	0.73
Business	14 (27.5%)	37 (72.5%)	0.63 (0.33–1.24	0.603
Medical insurance				
Yes	21 (19.3%)	88 (80.7%)	1	
No	117 (39.8%)	177 (60.2%)	1.632 (1.21–2.63)	0.033

### Reasons for indulging in self-medication

The majority of those who practiced self-medication with antibiotics (53.6%) gave reasons for the practice as to reduce medical cost, 26.2% said that there are long delays in health facility, while 11.6% did so because of a busy day’s program (Table 5).

**Table 5:** Reasons for indulging in self-medication

Reasons for SMP	Frequency	Percentage
Lack of clinicians	6	4.3
Busy day program	16	11.6
Cost cutting	74	53.6
Long delays in health facility	36	26.2
Previous experience of medical treatment of the same symptoms	6	4.3
Total	138	100

### Indication for SMP

**Fig. 5:**
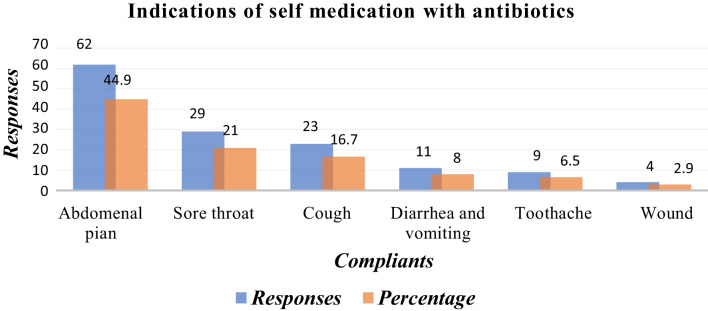
Complaints for SMP

Various respondents gave their complaints for taking antibiotics as follows: abdominal pain 44.9%, sore throat 21%, cough 16.7%, diarrhea and vomiting 8.0%, toothache 6.5%, and wound 2.9% (Fig. 5).

### Patients who were advised on self-medication with antibiotics

Few participants (21.1%) had been advised to take medication without prescription, while 78.9% were advised but did not self-medicate. Advice had no significant association with self-medication (*P* > 0.05) (Table 6).

**Table 6:** Respondents advised on SM

Advised	SM	Not SM	*N*	*Χ*^2^	*P* value
Yes	23 (21.1%)	86 (78.9%)	109	0.028	0.7882
No	115 (39.1%)	179 (60.9%)	294		
Total	138	265	403		

### Respondents’ sources of advice for self-medication

Those advised to take an antibiotic the majority of the respondents (47.4%) said their source of advice was from a colleague, 28% from health workers, 12.3% from a relative (Table 7).

**Table 7:** Sources of advice for SMP

Sources of advice	Frequency	Percentage
Colleague	27	47.4
Health worker	16	28.0
Relatives	7	12.3
Neighbour	7	12.3
Advertisement	0	–
Total	57	100

### Commonly used antibiotics in SMA

**Fig. 6:**
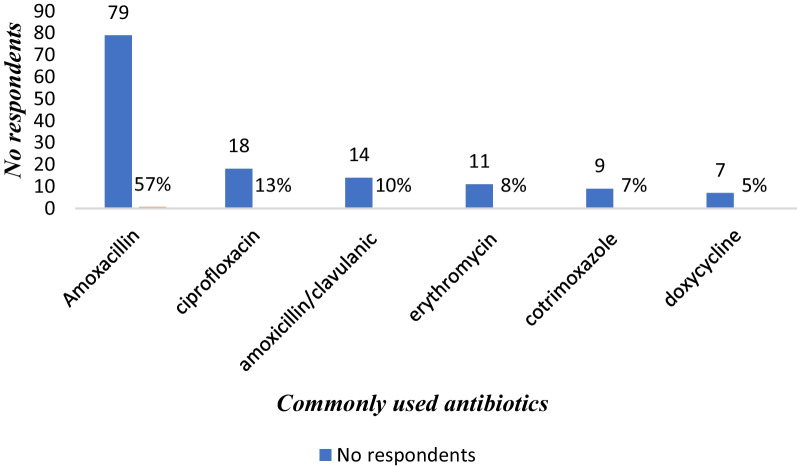
Antibiotics used in self-medication

The respondents were asked to mention the antibiotics that they had used without prescription. A list of antibiotics was provided to help the patients recall. The commonly used antibiotics are amoxicillin (57%), ciprofloxacin (13%), amoxicillin/clavulanic (10%), erythromycin (8%), cotrimoxazole (7%), and doxycycline (5%) (Fig. 6).

#### Sources of antibiotics

From Table 8, the majority of respondents who practiced self-medication with antibiotics got the drugs from community pharmacy (84.8%), others got drugs from health workers (8.7%), while 6.5% got the drugs from friends (Table 8).

**Table 8:** Sources of antibiotics

Sources	Frequency	Percentage
Community pharmacy	117	84.8
Health worker	12	8.7
Given by a friend	9	6.5
Shops	0	–
Total	138	100

### How respondents knew the dosage

As shown in the table above, it shows that 81.9% had enquired the dosage from the seller, while 15.9% used a previous prescription to know the dosage of the drug for self-medication (Table 9).

**Table 9:** How respondents established the dosage

Responses	Frequency	Percentage
Enquired from the seller	113	81.9
Used a previous prescription	22	15.9
Informed by a friend	3	2.2
Read on the packaging	0	–
Total	138	100

## Discussion

Patients who attended outpatient clinics were surveyed over the course of a month and a half. The prevalence of SMP was found to be 48.3% in the survey. Amoxicillin was the most widely used antibiotic for self-medication. Antibiotics were primarily obtained from local or community pharmacies. The primary motivation for using SMA was to save money. This study focused solely on antibiotic self-medication. Other drug classifications were excluded from the poll. The findings were still valid despite this constraint. To decrease the recall bias, a list of antibiotics was employed.

The majority of the respondents in this study were aged between 25 and 34 years at 31.1%. This was comparable with a study by Charles Kiragu Ngigi in Kenya, which had 27.7% of the respondents with the same age who practiced self-medication with antibiotics [32] and nearly also comparable with the study conducted in India, which found that 39% of respondents of the same age used antibiotics for self-medication [33]. According to this study, females account for 68.0% of practiced self-medication. In contrast [34], found that 44% of respondents in research in Saudi Arabia were female.

Self-medication with antibiotics was found to be 48.3%, which is lower than prior studies in Northern Nigeria, which found 56.8% and 50.3%, respectively [35, 36]. Self-medication with antibiotics was reported by 41.8% of respondents aged 18–24, which is comparable to data from [37] in Nigeria, who found 44% of respondents in the same age range. SMA and education had no meaningful relationship. Around half (46.7)% of those who self-medicate with antibiotics are divorced and 38.3% of those with secondary education. In this study, respondents who had not “gone to school” accounted for 25% of the total, implying that educated people accounted for 75% of the total, which is close to a study by Widayati et al. [38] that linked self-medication to a high percentage of education (78%). Other similar study reported in Nigeria, with just 14% of uneducated individuals self-medicating [39]. This link between self-medication and education could be due to the ease with which information can be obtained from various sources, including the internet.

Those who practiced self-medication with antibiotics had a smaller number of unemployed people (31%). This contradicts a study conducted by Askarian et al. [40], which revealed that 7.4% of the population was unemployed. SMA was practiced by 39.8% of persons who did not have health insurance, which was lower than the 46.3 percent reported by Askarian et al. [40] in Southern Iran. Self-medication with antibiotics is significantly connected with a lack of medical insurance.

Amoxicillin was the most often used antibiotic, with 79 (57%) of respondents using it. This was supported by studies conducted by Donkor et al. [41] in Accra, Ghana, and [42] in the United Arab Emirates, where amoxicillin had a high prevalence rate of 46% in both countries. According to a study conducted in Europe by Ali et al. [43], Greece has one of the highest outpatient antibiotic usage rates in Europe, with cephalosporins and macrolides being the most commonly prescribed antibiotics. The argument for the regular use of Amoxicillin was reinforced by the drug’s low cost around the world and its widespread prescription by healthcare practitioners, suggesting that it is well-known to the general people.

The antibiotics were obtained by a majority of responders (84.8%) from community pharmacy. This was contracted with a study conducted in Sudan, which found that 68.8% of the medications were obtained from a community pharmacy [44]. It is similar with other studies in India, with 79.5% of respondents getting their medications from pharmacies [45]. Other investigations in Palestine [20], Egypt [32], and other similar results [46]. The majority of respondents in all of these studies got their antibiotics from community pharmacies rather than through friends, health workers, or stores.

Antibiotics can be obtained from various sources: they are legally available over the counter, antibiotics originally prescribed by physicians can be saved and used without medical consultation, antibiotics can be obtained from friends or relatives, and they can be obtained via different sources.

## Limitations of the study

The research was done in a limited duration of 1 month. Patients who continued to practice the self-medication at home and did not visit the hospital during the period of study were not sampled. The identification of the actual antibiotic taken may not have been accurately recalled. The study was restricted to the practice of self-medication among adult patients, and patients below 18 years were excluded from the study. A list of antibiotics was provided to the patients to help them recall the drugs used.

## Recommendations

- **For policy makers**
  - Health education initiatives on antibiotic self-medication should be directed at persons per age, gender, educational levels, and the public at large.
  - Interventions aimed at minimizing antibiotic self-medication should focus on limiting access to antibiotics obtained without a prescription.
  - Antibiotics will not be sold over the counter without a prescription at community pharmacies.
  - The involvement of community pharmacists in reducing the prevalence of SMP should be significant.
- **For further researchers**
  More research is needed:
  - To determine whether widely self-medicated medications cause microorganisms to develop antibiotic resistance.
  - To determine the prevalence of antibiotic self-medication in children under the age of 18.
  - To investigate the public awareness and perceptions of antibiotic self-medication

## Conclusion

Antibiotic self-medication was prevalent among the study participants. Self-medication with antibiotics was 48.3 percent of the time, whereas general self-medication was 71%. Antibiotic self-medication is significantly associated with age, being a college or university student, and lack of health insurance. Self-medication with antibiotics is more common among adults aged 25–34 than in other age groups. The antibiotic amoxicillin was the most commonly self-medicated, followed by ciprofloxacin. Community pharmacies are a common source of antibiotics for self-medication. Self-medication with antibiotics, primarily amoxicillin and ciprofloxacin, was used to alleviate cough, abdominal pain, diarrhea, and vomiting. The majority of these drugs were gotten from a community pharmacy. In general, OPD patients appear to underestimate the possible hazardous effects of SMP. As a result, health officials should make an effort. As a result, health authorities such as the Drug and Therapeutic Committee, the Drug Regulatory Authority, hospital management, and other stakeholders should work together to ensure that antibiotics are used safely.

## Data Availability

Upon request to the corresponding author, all available supplementary information, including the data sets supporting the conclusions of this article, is made available.

## Abbreviations

FDA: Food and Drug Administration
FIP: Federation International Pharmaceutics
MOH: Ministry of Health
MOPC: Medical out-patient clinic
OPD: Outpatient department
OTC: Over the counter
PHs: Public Hospitals
RVU: Rift Valley University
SM: Self-medication
SMA: Self-medication with antibiotics
SMP: Self-medication practice
SOPC: Surgical outpatient clinic
SP: Sulfadoxine/pyrimethamine
SPHMMC: St. Paul’s Hospital Millennium Medical College
SPSH: St. Peter Specialized Hospital
TASH: Tikur Anbessa Specialized Hospital
WHO: World Health Organization
WSMI: World Self-Medication Industry
